# Assessment of pH-Responsive Ionisable Lipid Nanoparticles as Cisplatin Delivery Vehicles for Treating Cisplatin-Resistant Ovarian Cancer

**DOI:** 10.3390/pharmaceutics18050614

**Published:** 2026-05-18

**Authors:** Sarigama Rajesh, Gwo Yaw Ho, Ravindu Fernando, Poh Yi Gan, Jessica Wu, Jiali Zhai, Joshua D. Ooi, Calum J. Drummond, Nhiem Tran

**Affiliations:** 1 Molecular Assembly Laboratory, School of Science, STEM College, RMIT University, Melbourne, VIC 3000, Australia; s3720799@student.rmit.edu.au (R.F.); maggie.zhai@rmit.edu.au (J.Z.);; 2 School of Clinical Sciences, Monash University, Clayton, VIC 3168, Australiajoshua.ooi@monash.edu (J.D.O.); 3 Department of Oncology, Monash Health, Bentleigh, VIC 3165, Australia

**Keywords:** pH-responsive, lipid nanoparticles, platinum resistance, monoolein, cisplatin loading, PDX-mouse model, hexosomes, cubosomes, pH-trigger

## Abstract

**Background:** Platinum-based chemotherapy, including cisplatin and carboplatin, is widely used to treat various cancers, including ovarian cancer. However, its clinical application is limited by dose-limiting toxicities and resistance, with a poor 5-year overall survival rate for ovarian cancer (35–40%). In this study, we used ionisable lipids and developed pH-responsive lipid nanoparticles (LNPs) to address platinum-resistance in ovarian carcinoma. **Methods:** Cisplatin was loaded into three LNP systems containing monoolein (MO) and synthetic cationic ionisable lipids (OE-Mo, OA-Py, and OA-Pi) dispersed in Pluronic F-127 with 0.9% NaCl. Cisplatin-loaded LNPs (Cis-OE-Mo-NP, Cis-OA-Py-NP, and Cis-OA-Pi-NP) were characterised for size, zeta potential, and internal mesophase structure. Encapsulation efficiencies were determined via HPLC after removing free drug by ultrafiltration. In vivo efficacy was tested using cisplatin-resistant human patient-derived xenograft (PDX) models. **Results:** The LNPs were well dispersed with particle size of 219–250 nm and a drug loading of ~1.2 mg/mL. Encapsulation efficiencies were 62%, 59%, and 64%, for Cis-OE-Mo-NP, Cis-OA-Py-NP, and Cis-OA-Pi-NP, respectively. Small angle X-ray scattering (SAXS) results showed that the LNPs are pH responsive with structural transitions from a cubic to a hexagonal phase at an acidic pH. Among the tested formulations, Cis-OA-Py-NP resulted in the most significant reduction in tumour volume by ~60% compared to treatment with cisplatin alone. However, they also showed significant toxicity, including >10% weight loss and gross lung and kidney damage, as confirmed by histology. **Conclusions:** These findings highlight the potential of Cis-OA-Py-NP in reducing tumour volume but underscore the need for further optimisation to improve safety and therapeutic applicability.

## 1. Introduction

Cisplatin (cis-diamminedichloroplatinum II) is a platinum-based coordination complex with significant genotoxic activity [1]. It forms covalent cross-links with DNA, thereby disrupting replication and transcription. Clinically, cisplatin is a key first-line chemotherapeutic agent for multiple malignancies and is widely used in the management of solid tumours, particularly ovarian cancer [2].

Among gynaecological cancers, ovarian cancer accounts for the highest number of deaths and is commonly detected only once it has reached an advanced stage. Surgery, platinum-based chemotherapy, and Poly (ADP-ribose) polymerase (PARP) inhibitors (PARPi) are important initial treatment options for ovarian cancer [3]. Although cisplatin is effective in many patients, it is associated with dose-limiting side effects such as neuropathy and renal-related toxicities. Unfortunately, almost all cancers will eventually develop platinum resistance despite its initial efficacy. Ovarian cancer cells can develop drug resistance by several mechanisms, including reduced intracellular drug concentration, enhancement of detoxification, increased DNA repair and damage function, modification in the tumour microenvironment, and evasion of the host immune with dysregulation of apoptosis [4].

The non-selective distribution of the drug between normal and tumour tissues impacts dose-limiting side effects, including acute nephrotoxicity, myelosuppression, and neurotoxicity [5]. The therapeutic window is mainly limited by toxicity and further diminished by tumours that display intrinsic platinum resistance or acquire resistance during treatment [6]. Due to the aggressive nature of the high-grade epithelial ovarian cancer, the advanced stage of diagnosis and the inevitable emergence of drug resistance, with a 5-year overall survival rate disappointingly ranging between 35% and 40% [7]. The outcome for women with ovarian cancer, could be improved with a better drug delivery system to enhance the efficacy of platinum chemotherapy agents.

Various nanoparticle carriers have been investigated for cisplatin delivery, taking advantage of the high accumulation at the tumour site due to the enhanced permeability and retention (EPR) effect, which results from the leaky neovasculature and poor lymphatic drainage of tumour tissue [8]. Several formulations, such as polymeric micelles (NC-6004) [9], polymer conjugates (AP5280) [10], and liposomes (B103, SPI-077, and Lipoplatin) [11,12], have demonstrated promising results in preclinical studies. These formulations effectively met safety criteria but failed to improve efficacy over free cisplatin. However, many of these systems rely primarily on passive delivery and EPR-mediated accumulation, with limited responsiveness to the tumour microenvironment. In contrast, pH-responsive lipid nanoparticles capable of undergoing internal structural transitions under acidic conditions may provide an additional mechanism for controlled intracellular delivery and drug release. Further efforts are needed in the clinical development of cisplatin delivery systems designed to reach tumour cells and release their payloads locally to achieve optimal beneficial effects [10,11,12,13].

**Figure 1:**
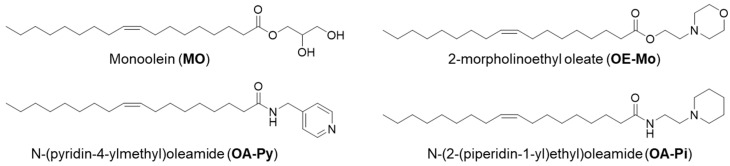
Structure of monoolein and ionisable aminolipids OE-Mo, OA-Py, and OA-Pi.

The current studies objective was to design cisplatin-loaded pH-responsive cubosomes and hexosomes using novel ionisable lipids to overcome cisplatin-resistance in an aggressive platinum-refractory ovarian cancer model, the ovarian carcinosarcoma. We have previously synthesised a library of nine novel ester aminolipids and formulated pH-responsive monoolein (MO)-based nanoparticles [14]. Some of these nanoparticles transitioned from hexosomes at neutral pH to cubosomes at lower pH (pH 4–7), often seen in the tumour microenvironment, making them prospective candidates for delivering chemotherapeutic drugs [14]. Further expanding the library, four amide aminolipids were synthesised and developed pH-responsive MO-LNP for drug delivery vehicles for solid tumours. The current study encapsulated cisplatin in three previously developed MO-LNP systems containing amino lipids 2-morpholinoethyl oleate (OE-Mo), N-(pyridin-4-ylmethyl)oleamide (OA-Py), and N-(2-(piperidin-1-yl)ethyl)oleamide (OA-Pi) (as shown in Figure 1). Cisplatin-loaded LNPs were evaluated for their physiochemical properties and compared to a drug alone control. Their in vivo efficacy was evaluated on tumour-bearing mice and compared to treatment with cisplatin alone. Tumour engraftment and progression have been measured and reported.

## 2. Materials and Methods

### 2.1. Materials

Quantities of 4-(2-Hydroxyethyl) morpholine (>99% purity), N-(3-Dimethylaminopropyl)-N′-ethyl carbodiimide hydrochloride (EDC), dimethyl aminopyridine (DMAP), dichloromethane (DCM), hydroxybenzotriazole (HOBt), n-hexane, ethyl acetate, sodium sulphate, sodium chloride, deuterated chloroform, Pluronic F-127, and cisplatin (95% purity) were purchased from Sigma-Aldrich (Melbourne, Australia). Ultrapure water (Milli Q, 18.2 MΩ·cm) was used for all solution preparations. Monoolein (MO) and oleic acid (OA) were sourced from Nu-chek-Prep, Inc. (Elysian, MN, USA) (>99% purity).

### 2.2. Synthesis, Purification, and Analysis of Ionisable Aminolipids

Synthesis of aminolipids was carried out using the procedures described in [14]. Nuclear magnetic resonance (NMR) imaging (^1^H) analysis confirmed the synthesised lipid structures (Figure S2). The chemical structures of 2-morpholinoethyl oleate (OE-Mo), N-(pyridin-4-ylmethyl)oleamide (OA-Py), and N-(2-(piperidin-1-yl)ethyl)oleamide (OA-Pi) are presented in Figure 1.

### 2.3. LNP Preparation

LNPs were prepared by a dry lipid film hydration method, adapted with minor changes from our previous work [15,16]. To prepare an empty nanoparticle (control without cisplatin), the dried lipid film was rehydrated with 1 mL of a Pluronic F-127 solution (2 mg F-127 in 1 mL of 0.9% NaCl solution). The resulting dispersion was then probe sonicated (Q Sonica) in pulse mode for 5 min to obtain a uniform suspension. Cisplatin-encapsulated LNPs were prepared by hydrating the dry lipids in an aqueous phase containing cisplatin. The aqueous phase was prepared by dissolving cisplatin (2 mg) in 1 mL of F-127 solution (2 mg in 1 mL of 0.9% NaCl). Table 1 represents the composition of each of the formulations prepared.

### 2.4. Particle Size, PDI, and Zeta Potential Measurements of LNPs

The hydrodynamic diameter, size distribution (reported as polydispersity index, PDI), and ζ-potential of the nanoparticles were characterised using a Zetasizer Nano ZS (Malvern Instruments, Malvern, UK). For size measurements, samples were diluted 1:10 in ultrapure water (MilliQ, Bayswater, VIC, Australia). ζ-potential was determined in 1.5 mM phosphate buffer (PBS) at different pH values, adjusted by addition of 0.01 M citric acid. Diluted samples (900 μL) were transferred to disposable cuvettes, and all measurements were conducted at 25 °C (n = 3) in accordance with the manufacturer’s instructions. Electrophoretic mobility was converted to ζ-potential by the instrument software using the Helmholtz–Smoluchowski equation [17].

### 2.5. Mesophase Identification

Nanoparticle phase behaviour was examined at the small- and wide-angle X-ray scattering (SAXS/WAXS) beamline of the Australian Synchrotron (ANSTO), using procedures adapted from our earlier work [14,15,16,17,18]. X-rays of wavelength λ = 1.128 Å (11.0 keV) with a typical flux of ~10^13^ photons/s were employed. A sample–detector distance of 1.6 m was selected, yielding a *q*-range of 0.01–0.5 Å^−1^. Two-dimensional scattering patterns were azimuthally integrated to generate one-dimensional intensity versus *q* profiles for phase assignment. Silver behenate served as the calibration standard. Each sample was exposed for 2 s, and no evidence of radiation-induced structural changes was observed.

The pH study was performed by mixing 40 μL of the nanoparticle with 40 μL of a buffer of the desired pH (citric acid/sodium phosphate system). The sample temperature was maintained at 25 °C using a circulating water bath during data collection.

### 2.6. Determination of Encapsulation Efficiency (EE%) and Drug Loading (DL)

**Figure 2:**
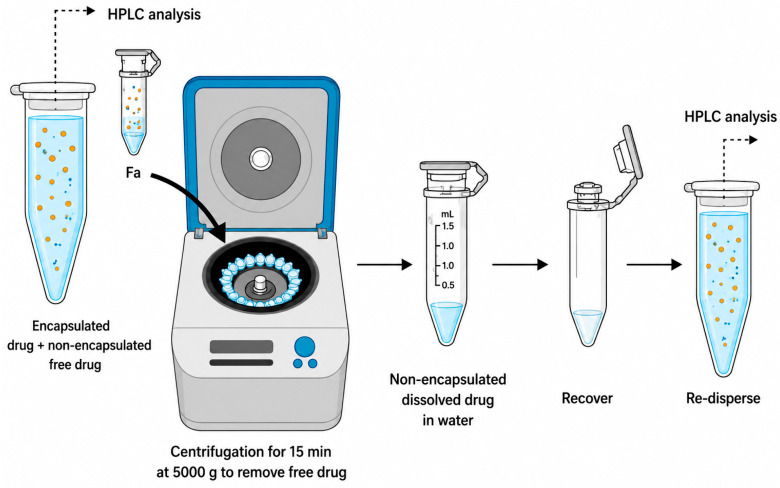
Representation of the ultrafiltration and centrifugation process followed in order to determine drug loading and entrapment efficiency.

Encapsulation efficiency (EE%) and drug loading (DL) were quantified after separation of non-encapsulated cisplatin using an ultrafiltration–centrifugation approach with Amicon^®^ centrifugal filters (Sigma-Aldrich (Melbourne, Australia)) (10 kDa molecular weight cut-off), as illustrated in Figure 2. Cisplatin-loaded LNP dispersions were transferred into the Amicon^®^ units and centrifuged at 5000× *g* for 15 min at 25 °C. The filtrate collected from the lower chamber contained the unencapsulated (free) drug; this fraction was brought back to the original volume prior to analysis.

Cisplatin content in the formulations before (W_i_) and after (W_f_) ultrafiltration/centrifugation was determined using a validated HPLC assay. Chromatographic separation was performed on an Agilent Zorbax SB-C18 column (Agilent, Santa Clara, CA, USA) (4.6 mm × 250 mm, 5 μm) equipped with an UV detector. The mobile phase consisted of acetonitrile and PBS (pH 5.0) at a 49:51 (*v*/*v*) ratio, delivered isocratically at 0.5 mL·min^−1^. The column temperature was maintained at 30 °C, and cisplatin was monitored by UV absorbance (detector wavelength as per the validated method; e.g., 210 or 232 nm). For analysis, 10 μL of formulation was diluted with 990 μL of a 50:50 (*v*/*v*) mixture of buffer and acetonitrile, and 20 μL of this solution was injected. Drug concentrations were obtained from a calibration curve.

EE% and DL were then calculated from W_i_ and W_f_ using the following relationships:

$$EE\%=\frac{Wf}{Wi}\times 100\;\mathrm{and}\;\mathrm{DL}=(\mathrm{Wi})-(\mathrm{Wf})$$

where W_i_ is the initial amount of drug in the formulation and W_f_ is the amount of free drug in the filtrate after centrifugation.

### 2.7. In Vivo Anti-Tumour Efficacy in Cisplatin-Resistant Tumour-Bearing Mice

**Figure 3:**
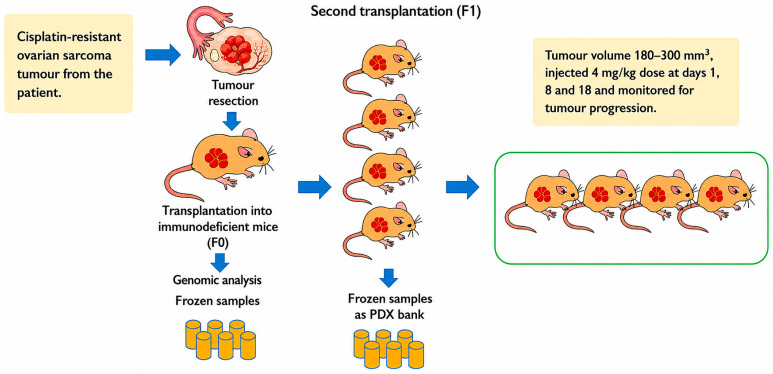
Graphical representation of PDX model followed for determining in vivo efficacy of cis-loaded LNPs, empty LNPs, cis-alone, and buffer treatments.

All animal experiments were performed at Monash University according to approved animal ethics (animal ethics approval granted for in vivo experiment (MMCB/2020/01/Ho) and human ethics approval had also been granted for the use of human specimens in the experiments (Monash University Human Research Ethics Committee—MUHREC/25377, dated 14 July 2020). Patient-derived xenograft (PDX) models were used to evaluate the efficacy of the cisplatin-loaded LNPs. PDX models have been identified as better representations of tumour heterogeneity and tumour microenvironment with retention of cellular complexity, cytogenetics, and stromal architecture, and are powerful tools for determining cancer characteristics, developing new treatments, and predicting drug efficacy [19]. Cisplatin-refractory ovarian cancer tumours from the patient were implanted subcutaneously in immunosuppressed NOD.CgPrkdc^scid^ H2-Ab1^em1Mvw^ H2-K1^tm1Bpe^ H2-D1^tm1Bpe^ Il2rg^tm1Wjl^/SzJ (NSG-(KbDb)^null^ (IA)^null^) NSG MHC^null^ mice (depicted in Figure 3). The tumour volume (mm^3^) was determined with the following equation:

Tumour volume (mm^3^) = π/6 × [larger diameter × smaller diameter^2^]

Once the tumour volume reached 180–300 mm^3^, treatments were administered at days 1, 8, and 18 at 4 mg/kg as an intraperitoneal injection. Tumour-bearing mice were randomised to cisplatin-loaded LNPs, treatments with cisplatin alone (cis-alone), and drug-free LNPs and buffer solutions (control). Mouse weight and tumour volume were measured twice a week for up to 120 days. The mice were culled once tumour volume reached 700 mm^3^ or if they showed signs of significant clinical deterioration.

The time to progressive disease (PD) was defined as the interval (in days) from treatment initiation to the point at which the mean tumour volume for a given treatment group exceeded the nadir value by more than 20%. The nadir was taken as the smallest mean tumour volume recorded after treatment commenced, or 180 mm^3^ if the minimum value was <180 mm^3^, as lesions below this size are difficult to measure reliably.

The time to harvest (TTH) was defined as the time (in days) from the beginning of treatment to the day of harvest at 700 mm^3^, and the median TTH was calculated and plotted using Kaplan–Meier curves (Graphpad Prism version 10). Tumour growth was monitored by measuring tumour volume three times per week and plotted as mean tumour volume ± SEM for each treatment group. Formal statistical comparison of treatment efficacy was performed using time to harvest (TTH), defined as the time required for tumours to reach the predetermined ethical endpoint requiring animal sacrifice. TTH was analysed using Kaplan–Meier survival curves, and significance between treatment groups was assessed using the log-rank (Mantel–Cox) test in GraphPad Prism (version 10). This endpoint was selected as it provides a robust and clinically relevant measure of tumour progression and treatment response in this model.

Paraffin-embedded kidney sections were evaluated semi-quantitatively for tubular injury. Tubular damage was assessed based on tubular epithelial cell loss, tubular necrosis, cellular debris accumulation, and tubular cast formation. The extent of injury was scored according to the percentage of affected tubules as follows: 0, normal; 1, <10–25%; 2, 26–50%; 3, 51–75%; and 4, >75% affected tubules.

## 3. Results and Discussion

### 3.1. Physicochemical Properties of Nanoparticles

The chemical structures of the synthesised ionisable lipids were confirmed by NMR analysis. The NMR analysis matched the analysis performed previously. Following the procedure explained in Section 2.3, LNPs were prepared using MO and three lipids: OE-Mo, OA-Py, and OA-Pi. Empty LNPs without cisplatin were named OE-Mo-NP, OA-Py-NP, and OA-Pi-NP, and cisplatin-loaded as Cis-OE-Mo-NP, Cis-OA-Py-NP, and Cis-OA-Pi-NP. The amount of cisplatin and Pluronic F-127 added to the nanoparticles (2 mg) was kept at 10 wt% to that of the total quantity of lipids in the LNPs. Table 1 shows the composition of the six LNPs.

The formulations were assessed for their particle size, PDI, drug loading (DL), and entrapment efficiency (EE%), and the results are presented in Table 2. All six formulations were well dispersed without visible phase separation or solid precipitations. The particle size of the LNPs prepared was 219–250 nm, while PDI was in the range of 0.15–0.22. These values are usually observed for non-lamellar LNPs [15]. Furthermore, cisplatin loading had a minimum effect on the particle size of the LNPs (Table 2).

The EE% and DL capacity of cisplatin-loaded LNPs Cis-OE-Mo-NP, Cis-OA-Py-NP, and Cis-OA-Pi-NP were also evaluated. As described in Section 2, the unencapsulated free drug was removed using the ultrafiltration and centrifugation method, and EE% was measured using HPLC. The calculated EE% for Cis-OE-Mo-NP, Cis-OA-Py-NP, and Cis-OA-Pi-NP was 62%, 59%, and 64%, respectively (Table 2), and there was no significant difference between their loading capacity. Compared to the study by Zhang et al. on similar MO-based cubosomes, our cis-loaded LNPs with MO and ionisable aminolipids possessed a drug concentration ~1.6–2 times higher [20]. Many factors influence the drug loading, including the lipid composition, the process of adding the drug and buffer, and the bilayer fluidity [21,22]. We have observed similar high drug loading of the water-soluble drug Fluconazole in our previous study [15]. The higher loading in this study may be due to aminolipids, which can act as ligands to replace chloride ions of cisplatin [23]. The higher loading in our studies may be due to the use of 0.9% NaCl solution, which will prevent the hydrolysis of cisplatin [Pt(NH_3_)_2_Cl_2_] in water [24].

**Figure 4:**
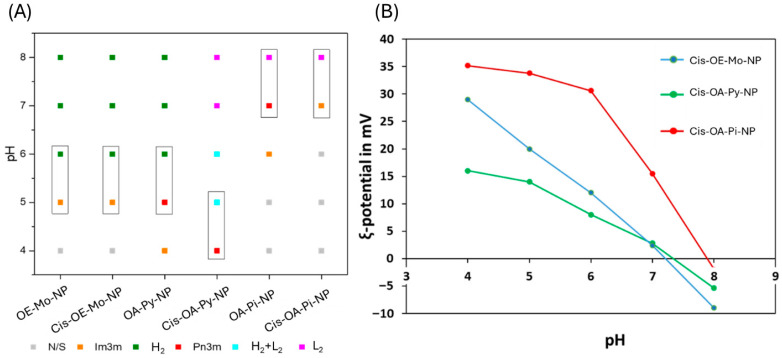
(**A**) A partial phase diagram for cisplatin-loaded LNPs and LNPs without the drug with respect to pH at room temperature. The boxes represent a phase transition to Q2 as the pH drops. (**B**) ζ-potential values for drug-loaded nanoparticles (Cis-OE-Mo-NP, Cis-OA-Pi-NP, and Cis-OA-Py-NP) under different pH values.

Synchrotron SAXS experiments were conducted with the cis-loaded LNPs to evaluate the effect of drug loading on the mesophase behaviour (Figure 4A). The phase behaviour of Cis-OE-Mo-NP at different pH values was very similar to that of the empty LNPs (OE-Mo-NP). Wherein, the Cis-OE-Mo-NP exhibited a H_2_ phase at neutral pH values and transitioned into an Im3m cubic phase with less curvature when the pH was changed from 6 to 5. The phase behaviour of Cis-OA-Pi-NP varied slightly from the empty LNPs, OA-Pi-NP. At pH 7, the L_2_ phase transitioned to an Im3m cubic phase instead of a Pn3m phase observed in the OA-Pi-NP.

On the other hand, Cis-OA-Py-NP differed from the control. The LNPs exhibited L_2_ phase at pH 7, as compared to an H_2_ phase for the drug-free OA-Py-NP. In the drug-free OA-Py-NP, a transition from the H_2_ phase to a Pn3m cubic phase occurred when the pH changed from pH 6 to pH 5. In Cis-OA-Py-NP, the coexistence of two phases (L_2_ + H_2_) was observed at pH 5.0, and a complete transformation to a Pn3m cubic phase occurred at pH 4. Cisplatin [Pt(NH_3_)_2_Cl_2_] contains an ionisable NH_3_ group, and several past studies have shown that encapsulated ionisable drug molecules can influence the mesophase structure of LNPs [25,26]. A recent study by Azmi et al. has also seen cisplatin-induced morphological alterations in the phospholipid-containing phytantriol system, wherein the encapsulated cisplatin has been localised in the internal H_2_ nanostructure, leading to an enlargement of its hydrophilic nanochannels [27]. The change can also be viewed from the ligand interaction of cisplatin with the aminolipid. The headgroup of OA-Py contains a pyridine moiety (Figure 1), and many studies in the past have shown that cisplatin can undergo ligand substitution with pyridine nitrogen atoms [23,28]. Cisplatin, being soluble in water, resides in the aqueous channels and can interact with the pyridine headgroup of OA-Py. An amount of OA-Py will participate in complexation with the drug, resulting in the phase transition pH (from L_2_ to Q_2_) occurring at one pH unit lower than the empty OA-Py-LNP.

The ζ-potential values for Cis-OE-Mo-NP, Cis-OA-Pi-NP, and Cis-OA-Py-NP were measured at different pH using a Zetasizer instrument (as described in Section 2). The measured ζ-potential values are plotted against pH and are presented in Figure 4B. At neutral pH, the ζ-potential values of the Cis-OE-Mo-NP, Cis-OA-Pi-NP, and Cis-OA-Py-NP were 0.4 mV, 15.6 mV, and 1 mV, respectively. Interestingly, Cis-OA-Pi-NP had a high value of ζ-potential (15.6 mV) at neutral pH; this may be due to the apparent pKa of the added ionisable lipid in the LNP [26]. As the pH of the system decreases, the ζ-potential values increase for all the tested LNPs. The observed increase in ζ-potential values is due to the presence of ionisable aminolipids, which are weak bases, and proton-induced ionisation results in the rise in ζ-potential values.

The pH-responsive behaviour of the present LNPs can be interpreted using the critical packing parameter (CPP), defined as $v/a_{0}l$, where v is the hydrophobic chain volume, $a_{0}$ is the effective headgroup area, and l is the hydrophobic chain length. The aminolipids used in this study are weak bases whose headgroups contain a nitrogen atom; as pH decreases, these headgroups become increasingly protonated, depending on their apparent pKa within the lipid assembly. This protonation increases electrostatic repulsion between adjacent headgroups, expands the effective headgroup area, lowers the CPP, and consequently alters the preferred interfacial curvature. This observation resembles our previously developed antifungal drug Fluconazole-loaded LNPs [15]. These changes provide a mechanistic basis for the pH-dependent mesophase transitions observed in the SAXS experiments. Differences in the headgroup chemistry of OE-Mo, OA-Py, and OA-Pi are therefore expected to influence ionisation behaviours, internal nanostructures, and the retention/release characteristics of cisplatin. While direct release studies were not performed in the present study, the structural and ζ-potential data support a formulation-dependent pH-responsive mechanism.

### 3.2. In Vivo Mouse Study

**Figure 5:**
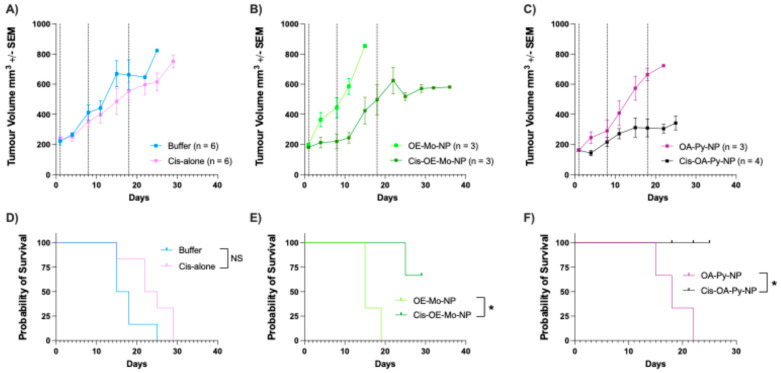
In vivo evaluation of cisplatin-loaded LNPs and control LNPs without the drug, along with cis-alone and buffer-alone treatments, using cisplatin-resistant PDX-tumour models in mice. For comparison of tumour growth in mice, broken vertical lines indicate treatment timing on day 1, day 8, and day 18. Measurements were taken for each treatment arm, n = 4 to 8. (**A**) Comparison of cis-alone to the buffer (saline) treatment. (**B**) Cis-OE-Mo-NP comparison to OE-Mo-NP. (**C**) Cis-OA-Py-NP comparison to OA-Py-NP. (**D**) Probability of survival for control groups after 30 days. (**E**) Probability of survival for Cis-OE-Mo-NP and OE-Mo-NP after 30 days. (**F**) Probability of survival for Cis-OA-Py-NP and OA-Py-NP after 30 days. (NS: not statistically significant; * *p* < 0.001).

To assess the in vivo efficacy of cisplatin-loaded LNPs in platinum-refractory ovarian tumours, tumour-bearing mice were randomised to empty LNPs, cisplatin-loaded LNPs, cisplatin alone, and saline injections. The treatment was conducted at a cisplatin dose of 4 mg per kg body weight. Empty LNPs were injected at the same lipid concentrations as cisplatin-loaded LNPs. Injections were given intraperitoneally on days 1, 8, and 18. Following treatment, the mice were monitored twice a week for tumour volume. The growth trajectories of the engrafted tumours over time have been presented on a line graph as mean of tumour volumes (mm^3^) ± SEM for all mice in their respective arms (Figure 5A–C). The present in vivo study was designed as an initial proof-of-concept evaluation using a single cisplatin dose across all formulations to enable direct comparison of formulation performance. However, this design does not allow a full assessment of dose-dependent efficacy and toxicity.

Cis-OA-Py-NP was the most effective therapy, demonstrating more than 60% reduction in the tumour volume compared to cis-alone (Figure 5C). Cis-OE-Mo-NP also showed in vivo efficacy in controlling tumour growth with 25–30% reduction in the tumour volume (Figure 5B). The reduction in tumour volume significantly prolonged the median TTH for the tumour-bearing mice group receiving the cis-loaded LNPs compared to the arms receiving cisplatin alone and PBS (as controls). These resulted in increases in the median TTH by 60% for the Cis-OE-Mo-NP arm and 100% for the Cis-OA-Py-NP arm, compared to the control arms (Figure 5E,F).

These findings were notable because the PDX tumours had been confirmed to be refractory to the platinum agent cisplatin, with treatment at the maximum tolerated dose (MTD) showing no impact on tumour growth. We hypothesised that achieving in vivo regression of these tumours would require a much higher intratumoral concentration of cisplatin to overcome the intrinsic tumour mechanisms of platinum resistance. Therefore, the in vivo tumour-killing efficacy of cis-loaded LNPs was likely owing to the ability of pH-responsive LNPs to deliver and concentrate cisplatin within the tumour micro-environment.

**Figure 6:**
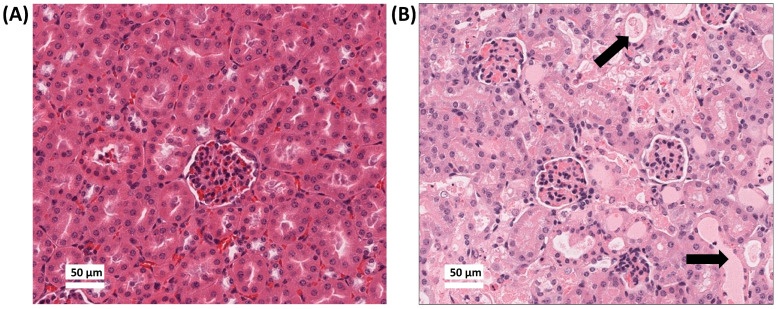
Haematoxylin and eosin (H&E) staining results of kidneys in mice treated with empty OA-Pi-NP (**A**) and Cis-OA-Pi-NP (**B**). Arrows show acute tubular necrosis (ATN) injury (×40).

The Cis-OA-Pi-NP treatment arm was terminated early due to reported toxicities after the first injection. All the tumour-bearing mice randomised to this arm appeared to be extremely scruffy and developed respiratory distress within 1–2 days after the first dose. All mice were euthanised after 2 days post treatment. To evaluate toxicity, a full warm autopsy was performed on these mice with kidney, liver, pancreas, lung, heart and brain collected for histologic assessment. Gross kidney abnormalities were observed in mice receiving Cis-OA-Pi-NP. The histology results demonstrated that the kidneys of OA-Pi-NP receiving mice appeared normal (Figure 6A). In contrast, there were extensive signs of acute tubular necrosis (ATN) found in the kidneys of mice receiving Cis-OA-Pi-NP (Figure 6B).

The severe acute toxicity observed for Cis-OA-Pi-NP is likely to reflect multiple formulation-dependent factors rather than a single mechanism. One possible explanation is greater instability of this formulation under physiological conditions, which may promote premature drug leakage after administration. In addition, SAXS analysis showed that Cis-OA-Pi-NP exhibited a cubic internal structure at neutral pH, and this type of mesophase has been associated with more rapid escape of water-soluble cargo through interconnected aqueous channels. The comparatively higher positive ζ-potential of Cis-OA-Pi-NP at neutral pH may also alter biological interactions in vivo and contribute to its adverse toxicity profile. These observations suggest that future optimisation should focus on improving formulation stability, reducing premature release, and refining lipid composition before further in vivo evaluation

To further assess renal toxicity, kidney injury was evaluated semi-quantitatively using paraffin-embedded kidney sections. Tubular injury was scored based on tubular epithelial cell loss, tubular necrosis, cellular debris accumulation, and tubular cast formation. Scores were assigned according to the percentage of affected tubules: 0, normal; 1, <10–25%; 2, 26–50%; 3, 51–75%; and 4, >75%. Empty nanoparticle-treated mice showed minimal tubular injury, with a mean score of 0.4 (n = 3), whereas cisplatin-loaded nanoparticle-treated mice exhibited severe tubular injury, with a mean score of 3.9 (n = 3).

Clinically, cisplatin is known for causing renal damage. The main reason for Cis-OA-Pi-NP toxicity is attributed to the preferential accumulation of cisplatin in the kidneys. Cisplatin induces the production of reactive oxygen species (ROS), which leads to mitochondrial and non-mitochondrial pathways of apoptosis and necrosis [2]. ROS also cause acute inflammation and cell infiltration. Histological analysis also confirms a large amount of protein casts within these renal tissues, which can be caused by a significant reduction in blood flow to the kidney. The reduction in blood flow can be due to the plasma fluid entering the renal interstitium [29].

Another possible explanation for the renal toxicity of Cis-OA-Pi-NP is the potential sudden release of high-concentration cisplatin from the cubosomal formulation, and the higher positive charge associated with the LNP. As observed by the SAXS study, Cis-OA-Pi-NP exhibits cubic structure at pH 7, and several studies have shown that water-soluble drugs tend to leak out of cubosomes in a “burst release” manner when the cubosomes change to a cubic structure phase [30,31]. The cubic phase formation results in two aqueous channels that interdigitate throughout the internal nanoparticle matrix, and these aqueous channels may provide the route for the drug to escape [30]. In contrast, the H_2_ phase maintains closed aqueous compartments, making it harder for the encapsulated drug to escape [31]. Cubosomes may also disintegrate under disintegration circumstances due to certain plasma interaction leading to a sudden release of the payload after injection [32].

**Figure 7:**
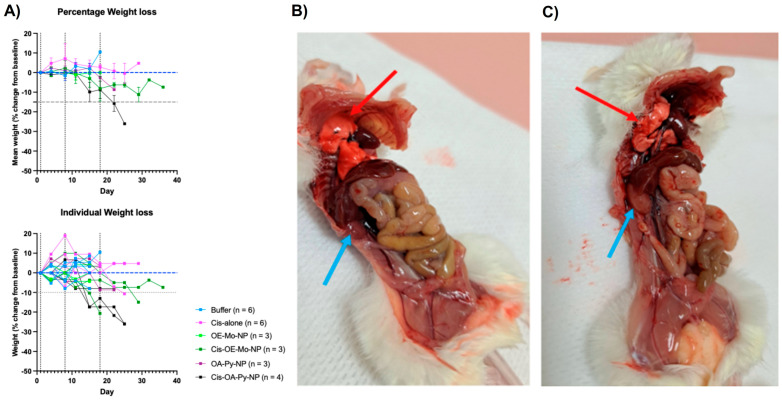
(**A**) Weight changes in the tumour-bearing mice measured after treatments. Measurements were done as mean percentage change from the original weight. The blue dashed horizontal line indicates baseline body weight (0% change), while the grey dashed horizontal line indicates the 15% weight-loss threshold. Vertical dotted lines indicate treatment administration time points. (**B**) Autopsy conducted on Cis-OA-Py-NP-treated mouse. Red arrow indicates mushy and damaged lungs and blue arrow indicates pale kidney with lost firmness. (**C**) Autopsy conducted on control OA-Py-NP-treated mouse. Red arrow indicates healthy lungs and blue arrow indicates unharmed kidney.

Cis-OA-Py-NP treatment resulted in significant mouse weight loss due to renal and lung damage. One of the long-term impacts of cisplatin treatment is severe cachexia, both in mice and humans [33]. This was observed in mice receiving cisplatin-loaded LNPs despite showing promising tumour growth inhibition. During a monitoring period of 40 days, the weight of the mice in all experiment arms were measured and presented in Figure 7A as the mean percentage change from the original weight. The results showed that tumour-bearing mice treated with Cis-OA-Py-NP exhibited significant body weight loss of >25% from baseline up to 30 days following treatment. The percentage weight losses in mice from the arms receiving buffer, cisplatin alone, or empty LNPs were not significant. This observation suggested that cisplatin LNPs can cause treatment-related toxicities leading to weight loss in these mice. In support of these findings, in vitro toxicity studies demonstrated that cisplatin-loaded nanoparticles were indeed more toxic to both A2780 and A2780cis (cisplatin-resistant) cells compared to free cisplatin and empty nanoparticles (Supplementary Figure S1 and Table S1). Notably, the inclusion of OA-Py appeared to further enhance the cytotoxicity of the nanoparticles.

Autopsies of Cis-OA-Py-NP-treated mice (Figure 7B) indicated gross damage to the mice kidneys and lungs after treatment, while the liver, spleen, pancreas and heart were spared. As a control, the autopsy results of the empty LNPs, OA-Py-NP, showed no gross damage to their kidneys and lungs (Figure 7C).

It is important to note that the empty control LNPs (OA-Pi-NP, OA-Py-NP, and OE-Mo-NP) do not have any anticancer activities or any toxicity effect. Therefore, the kidney damage seen for Cis-OA-Py-NP was likely related to increased concentration of cisplatin (a known renal toxic agent) in the kidney. The observed lung damage was also observed, possibly because of the vasculature and basement membrane of the lung are similar to those of the kidney, and toxicity is due to the higher concentration of drugs in the two organs. The observed kidney and lung toxicities are consistent with altered tissue exposure to cisplatin-loaded LNPs, but not definitive evidence of selective accumulation.

This higher accumulation in the lung may be attributed to the positive charge associated with the LNPs as explained by Cheng et al. in their recent study [34]. Cheng et al. developed a Selective Organ Targeting (SORT) strategy by adjusting the surface charge of LNP, which allowed LNP to accurately deliver the cargo molecule to the lungs, spleen and livers of mice when administered intravenously [34]. In the study, Cheng et al. added positively charged and negatively charged SORT molecules as a supplemental component to the traditional LNPs used for RNA delivery. An increase in the addition of positively charged SORT molecules using (2,3-dioleoyloxypropyl)trimethylammonium chloride (DOTAP) resulted in tissue-specific mRNA delivery shift from the liver to the spleen to the lungs. Adding a permanent positively charged SORT molecule completely changed the delivery from the liver to the lungs. On the other hand, adding permanently negatively charged SORT molecules meant that the majority of mRNA delivery was found in the spleen [34]. It is possible that due to the presence of the ionisable lipid, the LNPs are partially positive charge, leading to accumulation in these organs.

The differences observed between the three cisplatin-loaded formulations highlight a clear structure–property–performance relationship. OE-Mo-containing LNPs showed pH-responsive behaviour with relatively moderate in vivo efficacy and a more favourable tolerability profile. OA-Py-containing LNPs exhibited altered phase behaviour in the presence of cisplatin, potentially reflecting interactions between the pyridine headgroup and the drug, and this formulation showed the strongest anti-tumour activity but also substantial toxicity. In contrast, OA-Pi-containing LNPs displayed a higher neutral-pH ζ-potential and severe acute toxicity, suggesting that headgroup chemistry and resulting nanostructural behaviour strongly influence biological performance.

A study by Summers et al. demonstrated that mast cells mediate cisplatin nephrotoxicity and renal injury, and confirmed the therapeutic potential use of the mast cell stabiliser sodium chromoglycate for the prevention of cisplatin-induced acute kidney injury [35]. Administering sodium chromoglycate along with the cis-loaded LNPs may be a viable path to reduce the renal toxicity.

## 4. Conclusions

In this study, we have shown that we can improve tumour growth inhibition using cisplatin loaded into pH-responsive LNP in a cisplatin-refractory ovarian cancer in vivo model. Cisplatin was loaded into three previously developed pH-responsive LNP systems containing aminolipids OE-MO, OA-Py, and OA-Pi (named Cis-OE-Mo-NP, Cis-OA-Py-NP, and Cis-OA-Pi-NP). The prepared LNPs were characterised for particle size, zeta potential, drug loading, and encapsulation efficiency. The particle size was 219–250 nm, with PDI of 0.1–0.22 and a high drug loading of ~1.2 mg/mL, making the LNPs suitable for further in vivo evaluation. The in vivo efficacy of cisplatin-loaded LNP was determined using an ovarian carcinosarcoma PDX model, wherein a cisplatin-resistant tumour from a patient was implanted subcutaneously in immunosuppressed NSG MHC^null^ mice. The tumour volume measurement indicated that among the tested formulations, Cis-OA-Py-NP resulted in the most significant reduction in tumour volume by ~60% compared to treatment with cisplatin alone.

While the cisplatin-loaded LNPs showed promise in controlling tumour growth, they also caused more than 10% weight loss in mice, while the autopsy results indicated lung and kidney damage. Even though preliminary results are encouraging, further work in terms of optimising the formulation, evaluating the drug release mechanism from the LNPs, and safety studies are required. Future work will focus on refining aminolipid compositions and dosing strategies to improve tolerability, alongside evaluating serum stability and minimising premature cisplatin leakage. In addition, mechanistic studies, including pH-dependent in vitro release profiling, will be conducted to elucidate formulation-dependent release kinetics. Biodistribution studies will also be essential to assess organ-specific accumulation in vivo, supported by dose–escalation and dose–response studies in healthy and tumour-bearing mice, to define the therapeutic window and optimise the balance between efficacy and safety.

## Figures and Tables

**Table 1:** Compositions of LNPs prepared with and without Cisplatin.

LNP	MO (mg)	Aminolipid	Aminolipid (mg)	Cisplatin (mg)	F-127 (mg)	0.9% NaCl Solution (mL)
OE-Mo-NP	14	OE-Mo	6	0	2	1
Cis-OE-Mo-NP	14		6	2	2	1
OA-Py-NP	12	OA-Py	8	0	2	1
Cis-OA-Py-NP	12		8	2	2	1
OA-Pi-NP	17	OA-Pi	3	0	2	1
Cis-OA-Pi-NP	17		3	2	2	1

**Table 2:** Physicochemical properties of empty and cisplatin-loaded LNPs. Experiments were performed in triplicate, and results are represented as mean ± standard deviation.

Nanoparticle	Particle Size (nm)	PDI	%EE	Cisplatin Concentration (µg/mL)
OE-Mo-NP	219 ± 3	0.15 ± 0.03	N/A	N/A
Cis-OE-Mo-NP	228 ± 3	0.14 ± 0.03	62 ± 6	1240 ± 74
OA-Py-NP	222 ± 5	0.17 ± 0.03	N/A	N/A
Cis-OA-Py-NP	220 ± 2	0.10 ± 0.03	59 ± 3	1180 ± 35
OA-Pi-NP	250 ± 3	0.22 ± 0.03	N/A	N/A
Cis-OA-Pi-NP	248 ± 2	0.20 ± 0.03	64 ± 5	1288 ± 65

## Data Availability

The original contributions presented in this study are included in the article/Supplementary Materials. Further inquiries can be directed to the corresponding author.

## Supplementary Materials

The following supporting information can be downloaded at: https://www.mdpi.com/article/10.3390/pharmaceutics18050614/s1. Figure S1: In vitro cytotoxicity of cisplatin-loaded nanoparticles, free cisplatin, and control nanoparticles in A2780 and A2780cis cell lines. Table S1: IC_50_ values of cisplatin-loaded nanoparticles, free cisplatin, and control nanoparticles in A2780 and A2780cis cell lines. Figure S2: Proton nuclear magnetic resonance (^1^H NMR) spectra of synthesised ionisable lipids (OE-Mo, OA-Py, and OA-Pi).
