# Supplementary material for: Assessment of pH-Responsive Ionisable Lipid Nanoparticles as Cisplatin Delivery Vehicles for Treating Cisplatin-Resistant Ovarian Cancer
Source: Pharmaceutics. 2026 May 18;18(5):614. doi: 10.3390/pharmaceutics18050614 (PMC13210445; doi:10.3390/pharmaceutics18050614)
Supplement: Supplementary file 1 [file pharmaceutics-18-00614-s001.zip › pharmaceutics-4241996-supplementary.pdf]

Assessment of pH-responsive Ionisable lipid nanoparticles as cisplatin delivery vehicles for treating cisplatin resistant ovarian cancer

Supplementary Materials and Methods

Materials

A2780 and A2780cis human ovarian cancer cell lines were obtained from the European Collection of Cell Cultures (ECACC, Salisbury, UK). RPMI-1640 media, fetal bovine serum (FBS), trypsin-EDTA and penicillin-streptomycin additives were purchased from Invitrogen (Grand Island, NY, USA). MTS Assay Kit (Promega, Madison, WI).

Methods

***In vitro* testing – Cytotoxicity & Cell Viability Assay**

Ovarian cancer cells were cultured in monolayer using RPMI 1640 media, supplemented with 10% FBS and 1% PS, and grown in at 37°C in a humidified 5% CO_2 environment. To evaluate the cytotoxicity of cisplatin-loaded LNP formulations, A2780 and A2780cis cells were treated with the specified LNP formulations, and cell viability was determined using an MTS assay.

A2780 and A2780cis cells were first seeded into 96-well plates with a density of 2 x 10^4 cells per well and cultured in 100 µL cell media at 37°C in the presence of 5% CO_2, overnight. After a 24-hour incubation, the cell media was aspirated and replaced with 100 µL of LNP-containing media at lipid concentrations in the range of 400, 200, 100, 50, 25, 12.5, 6.25 µg/mL (achieved via serial dilution).

The combined cells and LNP solution were incubated at 37°C for a further 24-hour period. Following overnight incubation, 40 µL of MTS assay solution was added to each well. The 96-well plates were placed on an orbital shaker for 5min and incubated for 4 hours at 37°C in the presence of 5% CO_2. The absorbance was read on the SpectraMax Paradigm Multi-Mode Microplate Reader (Molecular Devices, CA, USA) at an optical density of 490 nm (absorbance) and 600 nm for reference wavelength or background reading.

Supplementary Results

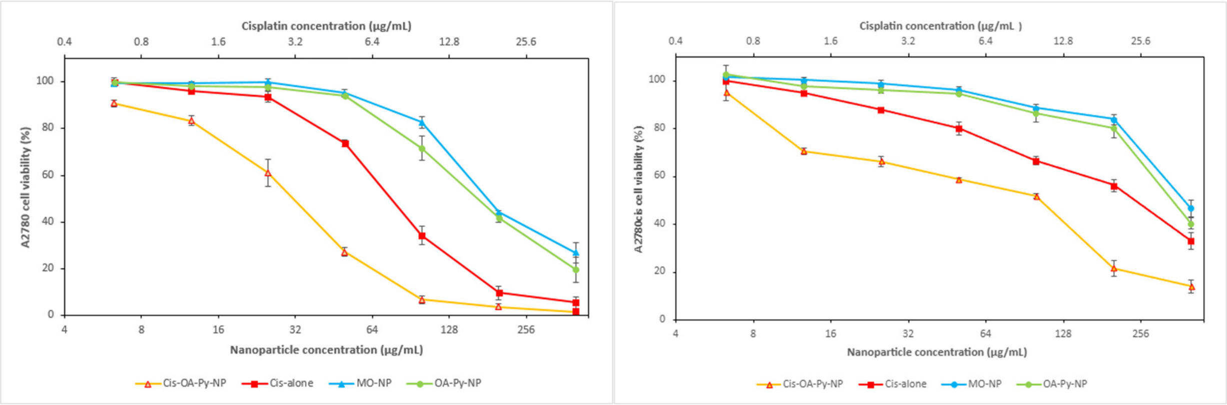

Figure S1. In vitro cytotoxicity of cisplatin-loaded nanoparticles in (A) A2780 and (B) A2780cis cell lines. Cell viability was assessed following 24 hours of treatment with Cis-OA-Py-NP, Cis-MO-NP, cisplatin-alone, and control nanoparticles (OA-Py-NP and MO-NP). Data are presented as mean ± standard deviation (n = 3).

Table S1. IC50 values for cisplatin-loaded nanoparticles, free cisplatin, and control nanoparticles in A2780 and A2780cis cell lines. IC50 values were calculated from cytotoxicity curves shown in Supplementary Figure S1. IC50 values represent the mean of the three independent experiments, standard deviation is not shown.

NP formulation	A2780 (ug/mL)	A2780cis (ug/mL)
Cis-OA-Py-NP	33	115
Cis-alone	74	265
MO-NP	192	400
OA-Py-NP	180	356

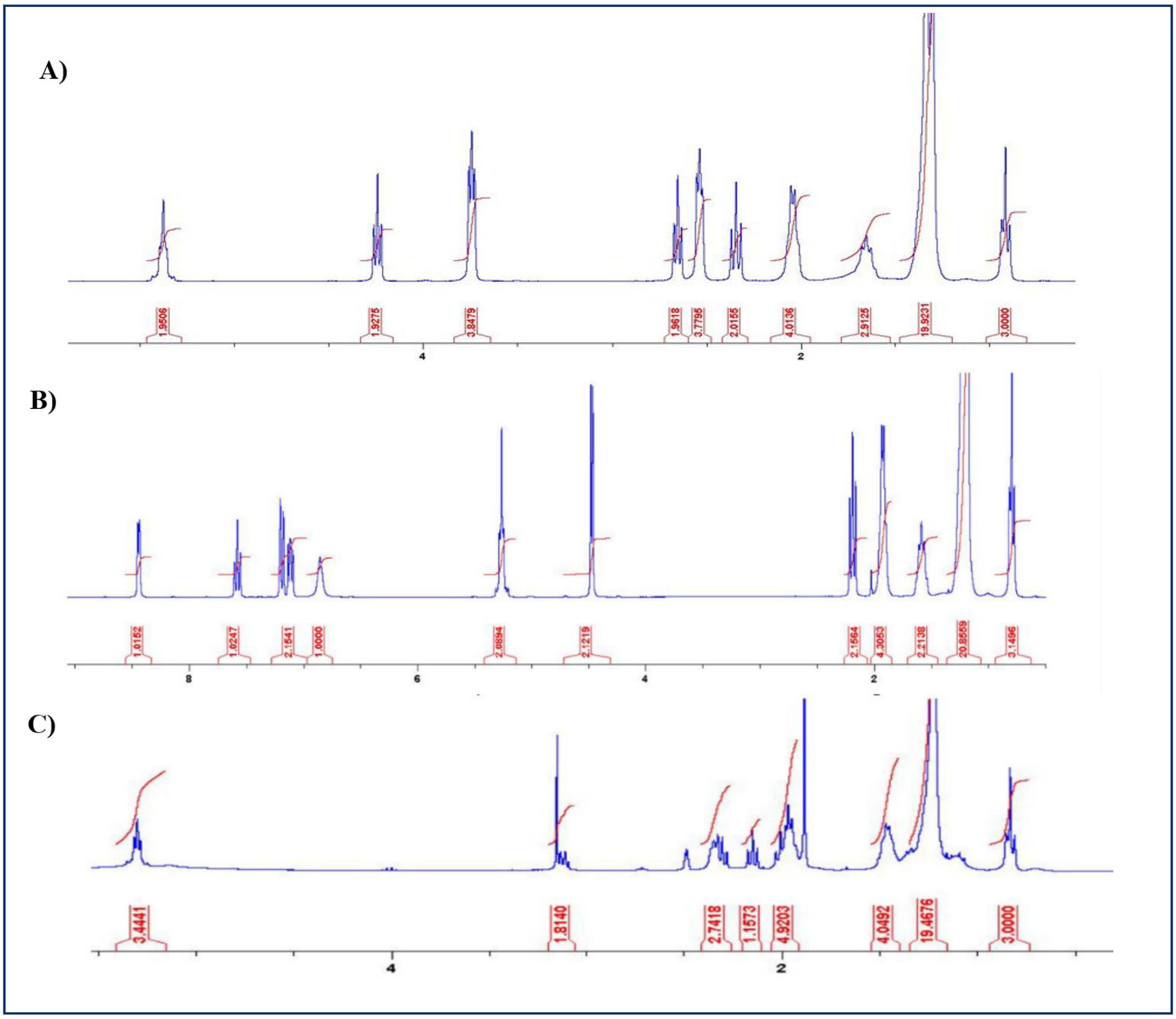

Figure S2: Proton NMR spectra for synthesized lipids A) OE-Mo B) OA-Py and C) OA-Pi
